# Trends towards an improved disease state in rheumatoid arthritis over time: influence of new therapies and changes in management approach: analysis of the EMECAR cohort

**DOI:** 10.1186/ar2561

**Published:** 2008-11-26

**Authors:** Isidoro González-Alvaro, Miguel Angel Descalzo, Loreto Carmona, the EMECAR Study Group

**Affiliations:** 1 Rheumatology Service, Hospital Universitario de la Princesa, c/Diego de León 62, Madrid 28006, Spain; 2 Research Unit, Fundación Española de Reumatología, c/Marques del Duero 5, 1°, Madrid 28001, Spain

## Abstract

**Introduction:**

The disease activity in patients with rheumatoid arthritis has improved during the past decade. The availability of new drugs and also a better assessment of the disease have been proposed to be responsible for this improvement. In the present work we estimate the effect of these factors on disease activity and function in patients with rheumatoid arthritis at the beginning of the new century.

**Methods:**

The Estudio de la Morbilidad y Expresión Clínica de la Artritis Reumatoide (EMECAR) cohort was assembled in 2000 from the random sampling of rheumatoid arthritis patients registered in 34 centers. The cohort was composed of 789 patients who underwent a baseline assessment plus four annual follow-up visits in which functional ability (Health Assessment Questionnaire score), the disease activity score obtained from 28-joint count with three parameters (DAS28-3) and radiological progression (Larsen score) were recorded. The effect of the calendar year on the DAS28-3, the Health Assessment Questionnaire score, and the Larsen score was obtained from adjusted models in which all treatments were included as dummy variables.

**Results:**

The effect of time as the β coefficient (95% confidence interval) for 2004, taking 2000 as a reference year, was -0.43 (-0.58 to -0.28) for the DAS28-3, 0.15 (0.07 to 0.22) for the Health Assessment Questionnaire score, and 4.4 (2.68 to 6.12) for the Larsen score. Treatment with new therapies, either leflunomide or TNF antagonists, increased in frequency from 1.1% (n = 8) in 2000 to 30.9% (n = 144) in 2004. Treatment with TNF antagonists (-0.28 (-0.5 to -0.05)) and with gold salts (-0.21 (-0.38 to -0.04)) was independently associated with a decrease in the DAS28-3 over time, whereas cyclosporin A treatment (0.45 (0.13 to 0.76)) was associated with an increase in disease activity.

**Conclusions:**

The mean disease activity of rheumatoid arthritis has improved from 2000 to 2004. An explanation is the introduction of new therapies, but not solely. Other factors related to the calendar year, plausibly a better management of available drugs, show a greater effect on improvement than the drugs used.

## Introduction

During the past decade, the number of therapeutic alternatives against rheumatoid arthritis (RA) has gratifyingly increased. Most of these new drugs belong to the so-called biologic agents, which have been developed against specific targets that play important functions in the pathogenesis of RA – namely, TNF, IL-1, CTLA-4, and CD20. Leflunomide (LEF) was introduced also in the past decade as a new nonbiologic disease-modifying antirheumatic drug (DMARD). TNF antagonists (aTNF) and LEF have demonstrated efficacy in randomized controlled trials, not only improving disease activity but also decelerating or arresting radiological damage [1,2]. When used outside trials, however, the effectiveness of new drugs may differ, since patients included in clinical trials are younger on average, have less comorbidity, and show greater disease activity than real-life patients [3]. In addition, drugs are prescribed according to strict protocols in clinical trials, while routine prescription is based not only on characteristics of the patients but also on physician's preferences [4,5].

While testing the hypothesis of a lower effectiveness of DMARDs and biologic agents in observational studies compared with clinical trials, we found that new drugs may have an impact – benefiting not only patients who are exposed to them, but also the nonexposed patients. The Estudio de la Morbilidad y Expresión Clínica de la Artritis Reumatoide (EMECAR) cohort was assembled before the widespread use of LEF and aTNF in Spain, during 1999 and 2000, and followed thereafter for four consecutive years [4], thus providing an adequate scenario to test hypothesis on new drugs. The present work describes what happened to RA patients followed up routinely in daily practice in terms of disease activity, disability and radiological progression in the time when LEF and aTNF were introduced.

## Materials and methods

The EMECAR cohort study has been previously described in detail [4,6]. The patient sample was formerly proven to adequately represent RA patients attending rheumatology tertiary hospitals in Spain, not very different from the mean RA patient followed up elsewhere [4,6].

### Sampling, recruitment, and data collection

All rheumatology clinics in Spain were invited to participate in EMECAR. Out of a total of 176 centers registered at the Sociedad Española de Reumatología database, 34 centers volunteered for participation (see Additional file 1). Participants had to send a file listing all patients ever registered at their clinics with a diagnosis of RA. Patients were randomly selected from these local databases, after checking for duplicates between centers. The selection complied with the Spanish regulations for Data Protection.

Participating rheumatologists were instructed to first confirm, on viewing the clinical records, the patients selected fulfilled the American College of Rheumatology 1987 criteria for the classification of RA [7]. Secondly, rheumatologists had to follow a contacting protocol that included three telephone calls on different days and at different hours, a search in mortality registries, and a letter to the address recorded in the database if needed. If a patient could not be reached after this protocol was followed, then the patient was discarded and replaced by another patient randomly selected in the same center. If a contacted patient did not want to enter the study, the patient was asked a short questionnaire to assess the reason for refusal and to determine basic sociodemographic and clinical characteristics.

All patients who entered the cohort signed a written consent form after being informed about the details of the study. The study protocol was reviewed and approved by the Research Ethics Committee of the Hospital Universitario de la Princesa, and follows all present ethical principles in clinical research.

The baseline visit of the EMECAR cohort took place between November 1999 and November 2000, although we will herein refer to this visit as the reference year 2000. Thereafter, four annual structured visits took place between 2001 and 2004 (see Additional file 2). The main objective of the cohort study was to estimate the expression of RA as well as to estimate the incidence of specific comorbidity in RA, and the prospective data collection included sociodemographic, clinical, therapeutic, laboratory, and radiological information. Participant rheumatologists were instructed to collect the data and were trained in the performance of joint counts and other measurements in a standardized fashion.

Data collected on DMARDs included their type, whether the drug was currently in use or had been used during the previous year, and the reason for any discontinuation. All data were obtained from the medical records and were confirmed with the patient during the study visits. Patients went through a complete physical examination and laboratory tests annually. The disease activity score was obtained from 28-joint counts using the formula with three parameters (DAS28-3) [8]. All patients completed the Spanish version of the Health Assessment Questionnaire (HAQ) to assess functional ability [9]. The radiological damage was assessed in X-ray scans of the hands and wrists biannually (2000, 2002, and 2004), which were read centrally by a trained radiologist blinded to the patient's record, and was scored by the Larsen method with the Scott modification (range 0 to 150) [10]. The radiologist performed an intraobserver reliability study on 20 randomized X-ray scans in which the codes had been changed to avoid recall. The intraclass correlation of the Larsen score between readings was 0.94 (95% confidence interval = 0.82 to 1.00).

Although the EMECAR cohort is formed by 789 patients, owing to different reasons we only have baseline values of the DAS28-3 in 735 patients, of the HAQ score in 777 and of the Larsen score in 678 patients. There were no relevant differences, however, in the characteristics of these patients with missing values compared with those studied (see Additional file 3).

### Statistical analysis

Mean differences between groups at baseline regarding continuous and nonparametrically distributed variables were analyzed using Student's *t *test and the Mann–Whitney *U *test, respectively. Association with categorical variables was tested with chi-square tests or Fisher's exact tests.

To determine the effect of the different DMARDs on the progression of the DAS28-3, the HAQ score, and the Larsen score we fitted a population-averaged model by weighted estimating equations nested by patient and visit, using the command *GENMOD *of SAS/STAT 8.2 for Windows (SAS Institute Inc., Cary, NC, USA). Weighted estimating equations are an extension of the generalized estimating equations in the presence of missing visits. This method consists of creating weights for each observation, at each time point, on the basis of previous observations and informative covariates. The weights represent the inverse probability of having dropped out and are then incorporated into the generalized estimating equations model. In our case, we used the fitted probabilities of having dropped out from a logistic regression with visit, sex, age and previous responses of the DAS28-3, the Larsen score and the HAQ score as independent covariates [11]. Additionally, patients with missing data were compared with the rest of patients in the covariates included in the analyses.

We tried different working correlations [12], such as independent, autoregressive (order 1), unstructured or exchangeable. We finally chose exchangeable among these competing correlation structures, using the smallest quasi-likelihood under the independence model information criterion values [13], measured in a bivariate analysis with year of visit as the only predictor. Actually, all of the above structures yielded similar results.

In addition to the calendar year and the DMARD prescription at each visit (gold salts, antimalarial, methotrexate (MTX), LEF, sulfasalazine cyclosporine A, aTNF and others), the following covariates were also analyzed: age at disease onset, age at baseline, gender, years of disease duration, presence of comorbidity (hypertension, diabetes mellitus, peptic ulcer, ischemic heart disease, heart failure, stroke, chronic obstructive pulmonary disease, neoplasms, liver disease, and depression), presence of any extra-articular manifestation (carpal tunnel syndrome, secondary Sjögren's syndrome, serositis, secondary clinical amyloidosis, rheumatoid vasculitis, eye disease, interstitial lung disease, rheumatoid nodules, Felty's syndrome, or anterior atlantoaxial luxation), rheumatoid factor positivity, serum hemoglobin, alkaline phosphatase, body mass index and the DAS28-3, the HAQ score and the Larsen score at baseline.

Bivariate analysis was performed with all factors and covariates, and then backward stepwise selection was applied in multivariate weighted generalized estimating equation models, starting with all variables that reached *P *< 0.2 in the bivariate analysis. The final models were reached by means of the quasi-likelihood information criterion [13] and we did not force any variable into the models (that is, we discussed whether gender should be included in models for the DAS28-3 but found there was also some collinearity with other variables, such as low hemoglobin).

In addition, we reran all analyses with the 448 patients (57.6%) who attended all visits; there were no relevant differences with the results obtained from the whole population.

## Results

### Treatment patterns

Patients who used aTNF or LEF at any time during follow-up were younger at baseline (2000), had an earlier RA onset, and presented more active disease than those who never used aTNF or LEF during follow-up (Table 1).

**Table 1:** Baseline (2000) characteristics of the patients in EMECAR

Characteristic	All studied patients	Ever used aTNF or LEF	Never used aTNF or LEF
*n*	789	210 (27%)	579 (73%)
Women	568 (72)	159 (76)*	409 (71)
Age (years)	61 ± 13	57 ± 12***	63 ± 13
Age at rheumatoid arthritis onset (years)	48 ± 15	44 ± 13***	50 ± 15
Rheumatoid factor-positive	592 (75)	167 (79)**	346 (70)
Any comorbidity	606 (77)	154 (73)	380 (77)
Any extra-articular rheumatoid arthritis	355 (45)	122 (58)*	226 (46)
Health Assessment Questionnaire score	1.2 ± 0.9	1.3 ± 0.8	1.2 ± 0.9
Disease activity score from 28-joint count with three parameters	4.1 ± 1.4	4.6 ± 1.4***	3.9 ± 1.3
Larsen score	54 ± 27	57 ± 24	53 ± 27

Data presented as *n *(%) or as mean ± standard deviation. Characteristics of those patients who used TNF antagonists (aTNF) or leflunomide (LEF) at any time of follow-up were compared with those of patients who did not. ***P < 0.001, **P < 0.01, *P < 0.05.

**Figure 1:**
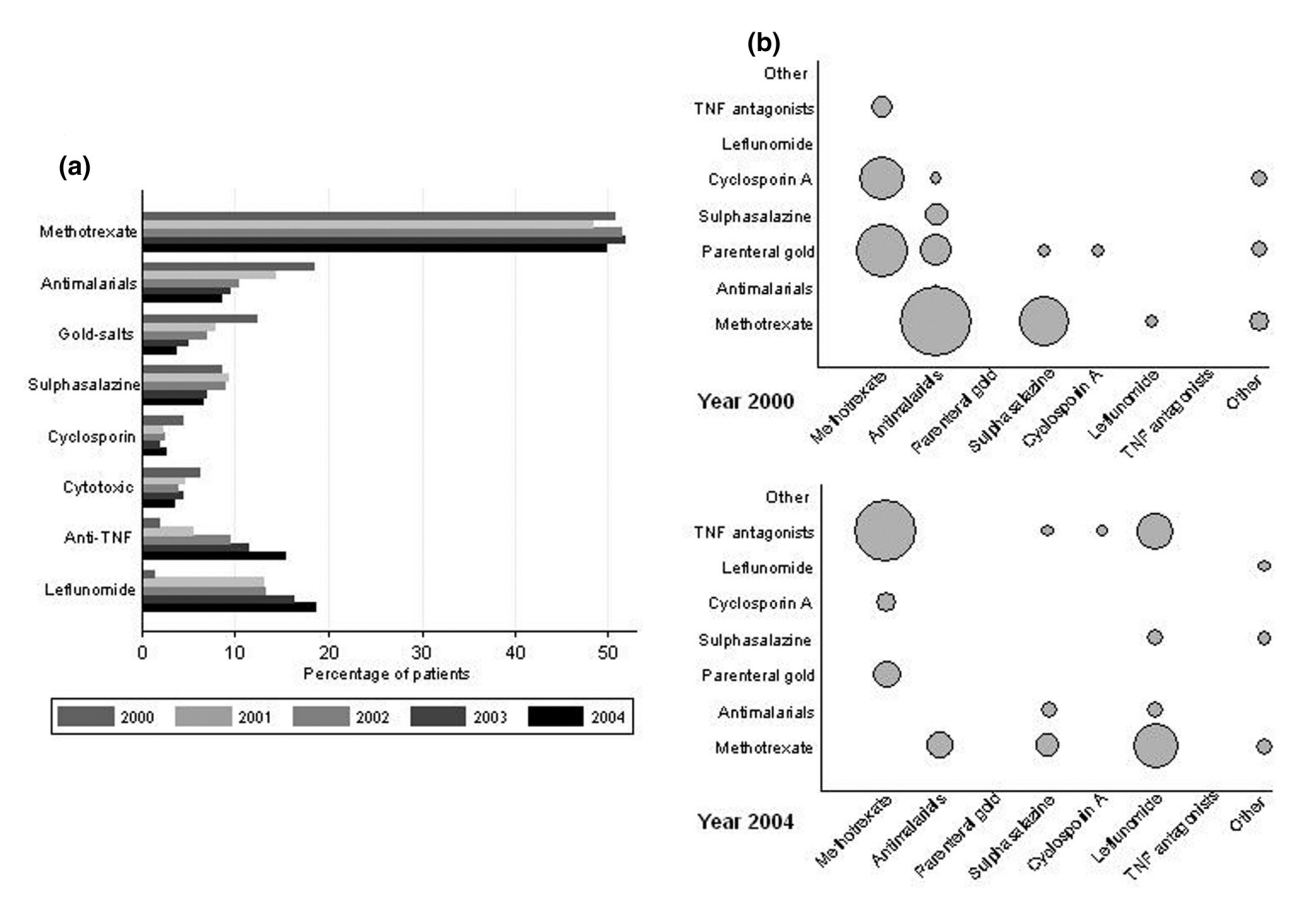
**Percentages of different systemic treatments and evolution of combination therapies**. **(a) **Percentage of patients treated with each of the different systemic treatments, by year of follow-up. **(b) **Evolution of combination therapies used in the EMECAR cohort. Circle diameters are proportional to the number of patients with the corresponding combination therapy, the smallest ones representing one patient (gold salts plus sulfasalazine in 2000) and the biggest circle representing 35 patients (methotrexate plus TNF antagonists in 2004).

Few patients were on aTNF and LEF in 2000, and those treated with them were either patients enrolled in clinical trials or were using these drugs compassionately. During 2001, both infliximab and LEF were authorized to be used in RA patients in Spain. Later on, etanercept (2002) and adalimumab (2003) were also marketed and approved, respectively. The introduction of aTNF and LEF into the market clearly correlated with the number of prescriptions year by year, from eight patients (1.8%) in 2000 to 143 patients (31.9%) in 2004 (Figure 1a). During 2004, 15.8% of patients were on an aTNF and 19% received LEF. Apart from MTX, which remained in use in around 50% of the patients, all other DMARDs experienced a decrease in their use over time, which was particularly evident in the case of gold salts.

The proportion of patients with no DMARDs decreased during follow-up, although not significantly (20%, 20%, 14%, 16%, and 16%, annually from 2000 to 2004). Both the percentages of patients on any DMARD monotherapy (from 57.8% to 59.8%, 2000 to 2004) or on combined therapy (from 22.6% to 24.2%, 2000 to 2004) increased, although none significantly. Of note, the drugs that were used in combination clearly changed during follow-up. In 2000, the drugs most frequently combined with MTX were antimalarials and gold salts, followed by sulfasalazine and cyclosporine (Figure 1b, upper panel). The scenario completely switched in 2004 towards combination therapy including MTX plus LEF or MTX plus aTNF (Figure 1b, lower panel).

We also observed that the combination of LEF and aTNF increased to become the third most frequent combination used in 2004 (Figure 1b, lower panel). Conversely, the most frequent combinations in 2000 – MTX plus antimalarials or MTX plus gold salts – became anecdotic in 2004. Triple therapy, namely MTX plus sulfasalazine plus antimalarials, supported by evidence [14], was only used in four patients during 2000 and used in three patients in 2004.

### Effect of time and treatment on disease

**Figure 2:**
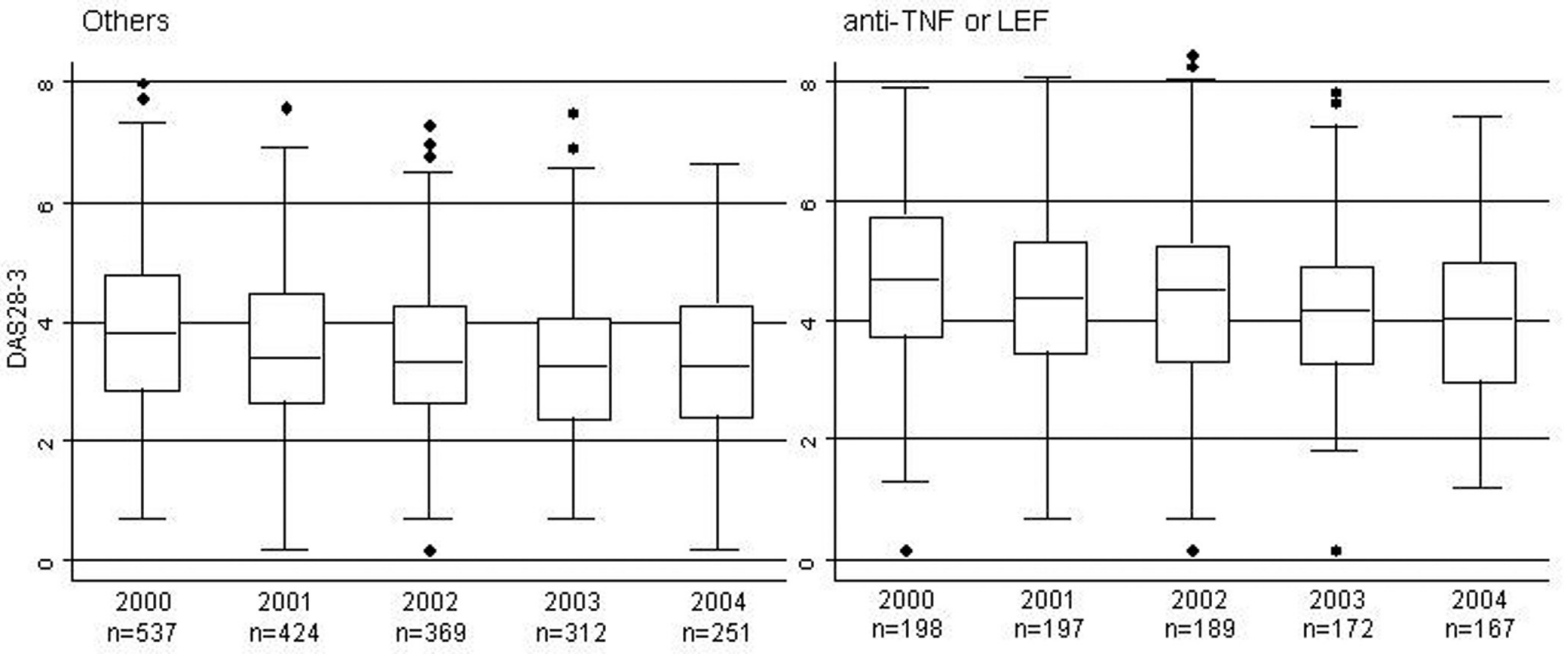
**Disease activity over time following treatment with new therapeutic agents or nontreatment**. Disease activity, as measured by the disease activity score from 28-joint count with three parameters (DAS28-3), over time in patients treated or not treated with new therapeutic agents, 2000 to 2004. LEF, leflunomide.

The median (interquartile range) of the DAS28-3 was 4.0 (3.0 to 5.1) in 2000, and this gradually decreased to 3.5 (2.7 to 4.6) in 2004. This decrease was also present when analyzing the patients who were treated and who were nontreated with new therapies separately (Figure 2). The HAQ score worsened slightly, from 1.125 (interquartile range = 0.50 to 1.875) in 2000 to 1.25 (interquartile range = 0.50 to 1.875) in 2004. The median Larsen score in hands was 48 (interquartile range = 34 to 71) in 2000, and increased to 55 (interquartile range = 37 to 70) in 2004.

To analyze the effect of time as well as the effect of each DMARD separately on the DAS28-3, the HAQ score, and the Larsen score over time, we first analyzed other predictors to adjust for as covariates in the weighted estimating equations models. The baseline DAS28-3 was associated with a significant progression of DAS28-3 and Larsen score (Table 2). In addition, extra-articular RA was also associated with higher DAS28-3 values and, conversely, higher levels of serum hemoglobin were associated with lower DAS28-3 values (Table 2). The baseline Larsen score was associated with a higher Larsen score during follow-up (Table 2). Being of an older age and presenting an increased HAQ value at baseline were both associated with worse progression of HAQ scores (Table 2). Regarding the effect of DAS28-3 on the HAQ, at each visit worse disease activity scores were associated with worse HAQ scores (Table 2).

**Table 2:** Variables associated with the evolution of the disease activity score from 28-joint count with three parameters (DAS28-3), the Health Assessment Questionnaire (HAQ) score, and the Larsen score in the EMECAR cohort

	DAS28-3		HAQ score		Larsen score	
			
	Bivariate	Multivariate	Bivariate	Multivariate	Bivariate	Multivariate
Female gender	0.57 (0.36 to 0.78)***	-	0.42 (0.29 to 0.55)***	-	6.49 (1.53 to 11.45)*	-
Age at baseline	0.001 (-0.006 to 0.007)	-	0.004 (0 to 0.008)*	0.002 (0 to 0.004)*	-0.19 (-0.34 to -0.03)*	0.009 (-0.025 to 0.043)
Age at disease onset	0.006 (-0.002 to 0.007)	0.002 (-0.002 to 0.007)	0.20 (0.016 to 0.024)***	-	0.57(0.42 to 0.73)	-
Disease duration	0.004 (-0.007 to 0.015)	-	0.017 (0.009 to 0.024)***	-	0.94 (0.55 to 1.34)***	-
Extra-articular rheumatoid arthritis	0.32 (0.15 to 0.4)***	0.16 (0.04 to 0.28)*	0.22 (0.13 to 0.31)***	0.03 (-0.02 to 0.09)	0.06 (6.35 to 11.76)***	-
Rheumatoid factor	0.19 (-0.03 to 0.41)	0.02 (-0.12 to 0.16)	0.14 (0.01 to 0.28)*	0.03 (-0.04 to 0.1)	7.96 (3.19 to 12.72)**	0.71 (-0.37 to 1.80)
Hemoglobin (g/dl)	-0.29 (-0.34 to -0.25)***	-0.19(-0.23 to -0.15)***	-0.1 (-0.12 to -0.07)***	-0.03 (-0.05 to -0.01)**	-0.95 (-1.61 to -0.28)**	-0.04 (-0.32 to 0.25)
Hypertension	0.05 (-0.11 to 0.21)	-	0.19 (0.11 to 0.27)***	0.05 (-0.01 to 0.11)	2.74 (0.4 to 5.08)*	-0.34 (-1.3 to 0.61)
Diabetes mellitus	-0.01 (-0.27 to 0.24)	-	0.07 (-0.08 to 0.22)	-	-1.87 (-6.54 to 2.8)	-
Hyperlipidemia	-0.14 (-0.31 to 0.03)	0.08 (-0.05 to 0.21)	-0.5 (-0.14 to 0.04)	-	-1.44 (-4.71 to 1.84)	-
Peptic ulcer	0.17 (-0.13 to 0.47)	-	0.15 (0.02 to 0.28)*	0.05 (-0.03 to 0.13)	1.30 (-3.06 to 5.66)	-
Myocardial ischemia	-0.05 (-0.33 to 0.23)	-	0.17 (-0.08 to to0.42)	0.06 (-0.09 to 0.21)	-0.49 (-6.56 to 5.58)	-
Heart failure	0.31 (0.04 to 0.59)*	0.06 (-0.21 to 0.33)	0.36 (0.21 to 0.50)***	-	-0.74 (-7.21 to 5.72)	-
Stroke	0.15 (-0.2 to 0.49)	-	0.02 (-0.27 to 0.3)	-	-8.94 (-13.79 to -4.09)***	-
Chronic obstructive pulmonary disease	-0.1 (-0.38 to 0.17)	-	0.01 (-0.13 to 0.14)	-	2.2 (-3.23 to 7.64)	-
Neoplasm	-0.29 (-0.61 to 0.03)	-	0.01 (-0.12 to 0.14)	-	-1.96 (-9.92 to 6.01)	-
Liver disease	0.11 (-0.1 to 0.33)	-	-0.2 (-0.11 to 0.08)	-	-3.63 (-9.01 to 1.76)	-2.57 (-5.07 to -0.07)*
Depression	0.27 (0.09 to 0.46)**	0.12 (-0.03 to 0.26)	0.15 (0.05 to 0.25)**	0.02 (-0.05 to 0.09)	-0.79 (-3.61 to 2.03)	-
Body mass index	0 (-0.02 to 0.02)	-	0.01 (0 to 0.02)	0 (-0.01 to 0)	-0.78 (-1.16 to -0.4)***	-0.16 (-0.65 to 0.33)
DAS28-3 (each visit)	-	-	0.17 (0.15 to 0.19)***	0.15 (0.13 to 0.17)***	0.37 (-0.23 to 0.98)	-
DAS28-3 (baseline)	0.62 (0.57 to 0.67)***	0.53 (0.48 to 0.59)***	0.24 (0.2 to 0.28)***	-0.1 (-0.13 to -0.07)***	6.79 (5.42 to 8.15)***	0.68 (0.26 to 1.11)**
HAQ score (each visit)	-	-	-	-	4.92 (3.61 to 6.24)***	-
HAQ score (baseline)	-	-	0.85 (0.82 to 0.88)***	0.82 (0.78 to 0.86)***	-	-
Larsen score (baseline)	-	-	-	-	0.97 (0.95 to 0.99)***	0.96 (0.94 to 0.98)***
Year						
∘ 2000	Reference	Reference	Reference	Reference	Reference	Reference
∘ 2001	-0.3 (-0.4 to -0.2)***	-0.23 (-0.33 to -0.13)***	0.01 (-0.03 to 0.05)	0.04 (-0.02 to 0.09)	NA	NA
∘ 2002	-0.32 (-0.43 to -0.21)***	-0.27 (-0.38 to -0.15)***	0.06 (0.01 to 0.11)*	0.07 (0.01 to 0.13)*	2.09 (1.32 to 2.86)***^°^	2.07 (1.1 to 3.04)***
∘ 2003	-0.47 (-0.58 to -0.36)***	-0.4 (-0.53 to -0.27)***	0.05 (0 to 0.11)	0.11 (0.04 to 0.18)**	NA	NA
∘ 2004	-0.5 (-0.63 to -0.37)***	-0.43 (-0.58 to -0.28)***	0.09 (0.03 to 0.15)**	0.15 (0.07 to 0.22)	4.18 (2.68 to 5.68)***	4.4 (2.68 to 6.12)***

Data presented as β coefficient (95% confidence interval). NA, not available. ***P < 0.001, **P < 0.01, *P < 0.05.

The effect of the calendar year on the DAS28-3, the HAQ score, and the Larsen score was obtained from adjusted models in which all treatments were included as dummy variables. The β coefficients and 95% confidence intervals during 2001 to 2004, taking 2000 as the reference year, are presented in the lower panel of Table 2. Disease activity decreased significantly compared with baseline year during follow-up.

**Figure 3:**
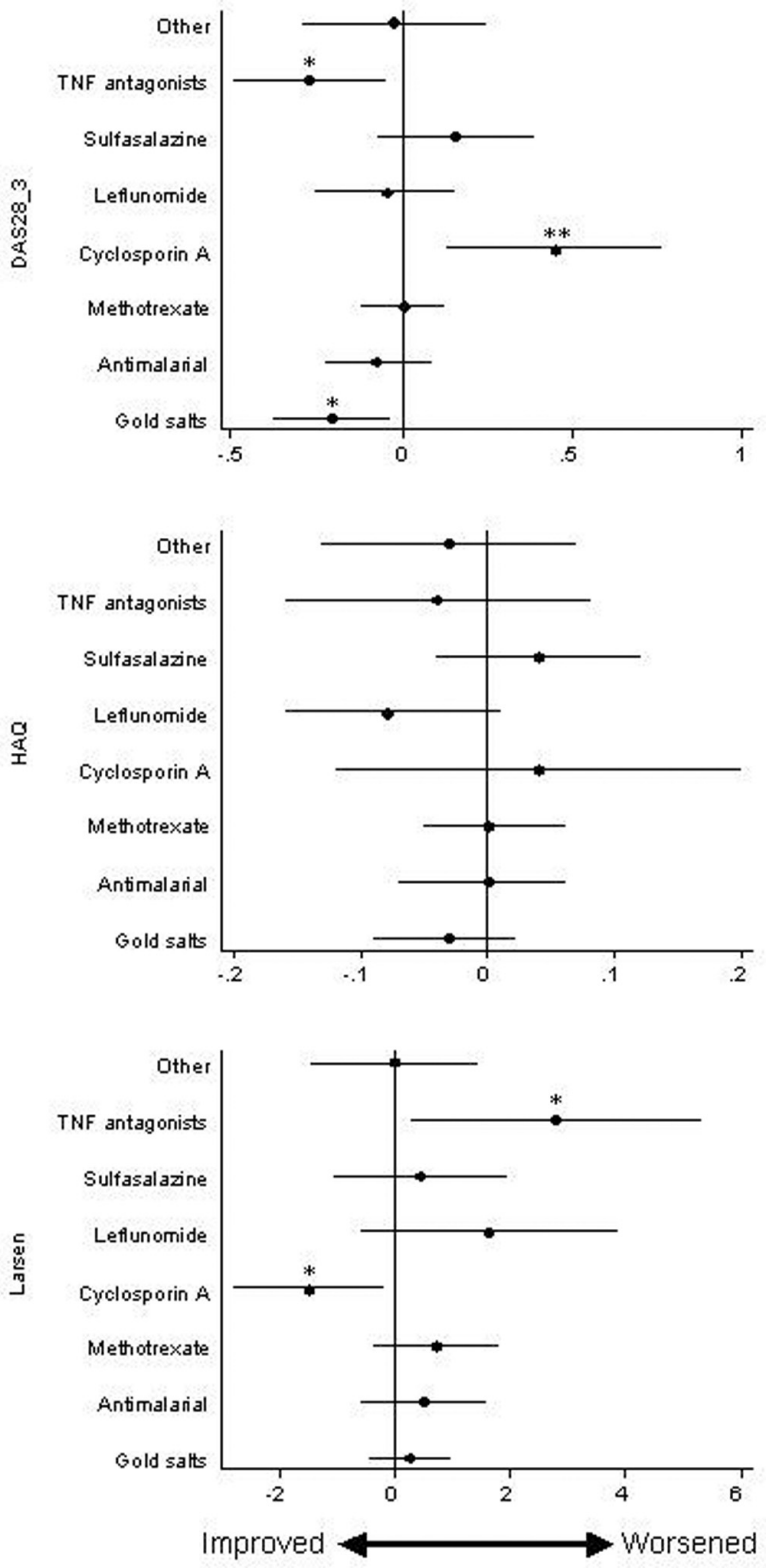
**Treatment effects over time on disease activity, function and disease progression**. Effect over time of different treatments on disease activity score from 28-joint count with three parameters (DAS28-3), function (Health Assessment Questionnaire (HAQ)), and disease progression (Larsen score). Pikes and lines represent the estimated β coefficients and 95% confidence intervals in the weighted estimating equations, adjusted by covariates and calendar year (see Statistical analysis). *P < 0.05, **P < 0.01.

The effect of individual DMARDs on disease activity, function, and damage over time was evaluated by including dummy variables for DMARDs in the final fitted models. Figure 3 shows the effect of individual drugs on the DAS28-3, the HAQ score, and Larsen score. As is shown in Figure 3 (upper panel), treatment with aTNF (β coefficient = -0.28 (95% confidence interval = -0.5 to -0.05), *P <*0.05) and with gold salts (β coefficient = -0.21 (95% confidence interval = -0.38 to 0.04), *P *< 0.05) was independently associated with a decrease in the disease activity, whereas cyclosporin A treatment (β coefficient = 0.45, 95% confidence interval = 0.13 to 0.76), *P *< 0.01) was associated with an increase in the DAS28-3 value. The remaining DMARDs did not seem to affect the DAS28-3 significantly. The whole effect of DMARDs on the HAQ score seemed to be the prevention of disability impairment – LEF was the only drug that tended to be associated with an improvement of this measurement, although it did not reach statistical significance (*P *= 0.073). Surprisingly, treatment with aTNF was associated with a significant radiological progression (Larsen β coefficient = 2.79 (95% confidence interval = 0.27 to 5.31), *P *< 0.05) and cyclosporin A treatment was associated with a significant improvement of this parameter (Larsen β coefficient = -1.51 (95% confidence interval = -2.8 to -0.23), *P *< 0.05).

## Discussion

The management of RA has experienced relevant changes during the past decade. The development of biologic therapies, as well as the rigorous clinical trials that have demonstrated their effectiveness, have probably contributed to this change. The most relevant finding of our work is that disease activity in RA has improved, independently of the availability of new therapies, in patients with severe disease, and also in patients with milder forms of the disease. Our work also proves that aTNF promote a significant improvement of RA disease activity in daily clinical practice in a similar way to randomized clinical trials. Disappointingly, we could not observe the amazing halt of radiological progression described in clinical trials.

Our data show that, once the effect of variables known to affect these outcomes was removed – such as age, rheumatoid factor, RA complications, disease duration, and comorbidity [15] – both disability and radiological progression worsened less than expected over time. Furthermore, when we included all DMARD treatments in the model, the effect of the calendar year on activity, disability and damage remained unchanged – thus reflecting a smaller effect of treatment than of other unmeasured variables that improved with time.

Our hypothesis is that this improvement may be associated with a more efficient management of RA by Spanish rheumatologists based at tertiary centers. As a matter of fact, during 1998 and 1999 most Spanish RA patients were on MTX but at insufficient doses [5] – the median of the highest MTX dose ever prescribed being 10 mg/week (interquartile range = 7.5 to 12.5 mg/week) [16]. The DMARD dose was not collected in EMECAR, but data from a national early RA register (Estudio de los factores pronósticos de enfermedad grave en la artritis reumatoide de reciente comienzo [Study of the prognostic factors in early arthritis]) show that in 2001 the median MTX dose was 7.5 mg/week (interquartile range = 7.5 – 12.5 mg/week) while in 2005 the median dose was 12.5 mg/week (interquartile range = 10 to 15 mg/week) (unpublished data). Two reasons may explain this change in the management of MTX. First, 90% of all Spanish rheumatologists in 1998 and 1999 had never collected the variables needed to estimate the DAS28-3 during daily clinical practice [17], therefore the systematic assessment of disease activity they had to perform per protocol in EMECAR led the rheumatologists to realize the poor control they had over their own patients. On the other hand, the finding may also reflect the experience of rheumatologists in numerous aTNF clinical trials, where fast MTX dose escalation was the norm [18,19].

Additional data support this change towards a more efficient treatment of RA in Spain. Throughout the follow-up of the EMECAR cohort, the percentage of patients without any DMARD declined while the frequency of patients on combined therapy increased. In addition, combination therapies with less supporting evidence, such as MTX plus antimalarials or MTX plus sulfasalazine [20], gradually disappeared as they were replaced by more aggressive combinations with better proven efficacy [18,19,21-25]. Finally, parallel to our findings, a more effective DMARD use has been recently demonstrated as the main reason for the decreasing progression of radiological damage in patients with RA over time [26].

In addition, our study provides some interesting findings about the result of gold salts and cyclosporin A prescriptions. We have observed that patients treated with parenteral gold salts showed a better DAS28-3 evolution together with an arrest in the evolution of the HAQ and Larsen scores. This finding was opposite to the impression of Spanish rheumatologists about this drug, since inefficacy was the most frequent reason to explain withdrawal of gold salts in EMECAR (see Additional file 4). Patients on gold salts might had a more benign disease course, however, and in the event they suffered a RA worsening or a flare up they were probably switched to another DMARD, instead of using gold as an add-on therapy.

Regarding cyclosporin A treatment, it was surprising that prescription of this drug was associated with the worst DAS28-3 evolution but, conversely, those patients displayed the best radiological evolution. The reason for this contradictory finding might be the well-known failure of cyclosporin A to improve the erythrocyte sedimentation rate [27,28]. Considering the large effect of the erythrocyte sedimentation rate on the disease activity score, the use of this score represents a handicap for the evaluation of the efficacy of cyclosporin A on disease activity. On the other hand, cyclosporin A inhibits the synthesis of IL-17 [29], which in turn promotes RANKL production. With this background it is possible that cyclosporin A decreases the RANKL/osteoprotegerin ratio and, therefore, can explain the remarkable effect of this drug in radiological progression, which has been also described by Jones and colleagues [30].

Regarding the impact of new therapies in RA, aTNF decreased the DAS28 score by, on average, 0.3 points. In opposition to the good concordance regarding the effect of aTNF on RA disease activity between data from clinical trials and our data from daily clinical practice, radiological progression was significantly worse in patients treated with these drugs. The most feasible explanation to this finding is that our cohort comprises mainly longstanding patients and aTNF were probably prescribed to those with more severe disease and higher Larsen scores at the beginning of the study (Table 1). In these conditions, it has been described that articular damage can progress in the absence of relevant disease activity [31]. Accordingly, with this disappointing radiological outcome in daily clinical practice, Listing and colleagues have reported that biologics provide higher remission rates than classical DMARDs, although in both cases the percentage of patients who reached this ideal state was very low [32]. In this regard, it has been described that patients in daily clinical practice are older and have more comorbidities than those included in clinical trials [3]. These characteristics may hinder the optimization of aTNF treatment and therefore may also underlie the results obtained in our study, which are less impressive than those reported in clinical trials. In addition, changes in tender joint counts may be hard to detect in older patients, as well as those with longer duration of disease. This may be due to the presence of residual damage or osteoarthritis, both resulting in low improvements in the disease activity score.

Considering all this information, we clearly need specific markers of RA severity that allow us to select adequate patients for early biologic treatment in order to improve their therapeutic response, as well as their functional outcome. These tools may also help to improve the cost-effectiveness of these drugs, avoiding unnecessary prescriptions.

## Conclusion

Our work shows that the mean disease activity of RA at tertiary hospitals in Spain has improved from 2000 to 2004. One explanation is the introduction of new therapies, since we have confirmed that the use of these drugs was associated with an improvement of the average disease activity score and of the HAQ score. Other factors related to the calendar year, however, show a greater effect than the drugs used on improvement. It is probable that a better management of available drugs, mainly MTX, has been learnt during the past decade along with the clinical development of most biologic agents, during which MTX has been used in a fast dose-escalation fashion. In addition, the systematic assessment of disease activity required in the EMECAR follow-up may have helped Spanish rheumatologists to realize that patients were not adequately controlled, thus leading to enhancement of patients' treatment.

## Abbreviations

aTNF: TNF antagonists; DAS28-3: disease activity score obtained from 28-joint count calculated using the formula with three parameters; DMARD: disease-modifying antirheumatic drug; EMECAR: Estudio de la Morbilidad y Expresión Clínica de la Artritis Reumatoide; HAQ: Health Assessment Questionnaire; IL: interleukin; LEF: leflunomide; MTX: methotrexate; RA: rheumatoid arthritis; RANKL: Receptor Activator for Nuclear Factor kappaB ligand; TNF: tumor necrosis factor.

## Competing interests

The EMECAR study was funded by the Spanish Society of Rheumatology, the Spanish Foundation of Rheumatology, and by an independent research grant from Aventis, formerly from Hoechst Marion Roussel. IG-Á has in the past 5 years received unrestricted research funding from Abbott Laboratories, Sanofi-Aventis and Bristol-Myers Squibb. All these research projects have no relation to the present work. LC and MAD have no competing interests.

## Authors' contributions

LC participated in the design of the study and the interpretation of data, and helped to draft the manuscript. MAD performed the statistical analysis and helped to draft the manuscript. IG-A participated in the design of the study and also in collection of the data at the Hospital Universitario de la Princesa, was involved in the interpretation of data and drafted the manuscript. All rheumatologists of the EMECAR group were involved in collection of data. All of the authors read and approved the final version of the manuscript.

## Supplementary Material

Additional file 1 A Word file listing the collaborators in the EMECAR study. Click here for file

Additional file 2 An Adobe file containing a figure that shows the flowchart of the EMECAR study, providing relevant information about the dropouts along the follow-up. Click here for file

Additional file 3 A Word file containing a table that presents the characteristics of the studied patients and the nonstudied patients in the multivariable analysis of each variable: disease activity, functional disability and radiological damage. Click here for file

Additional file 4 An image file containing a graph of reasons for discontinuation during follow-up among therapies. The smaller the space between levels of different variables, the greater the association between them. AM, antimalarials; aTNF, TNF antagonists; GS, parenteral gold salts; LEF, leflunomide; MTX, methotrexate; SSZ, sulfasalazine. Click here for file
