# Supplementary material for: Trends towards an improved disease state in rheumatoid arthritis over time: influence of new therapies and changes in management approach: analysis of the EMECAR cohort
Source: Arthritis Res Ther. 2008 Nov 26;10(6):R138. doi: 10.1186/ar2561 (PMC2656242; doi:10.1186/ar2561)
Supplement: Additional file 1 — A Word file listing the collaborators in the EMECAR study. [file ar2561-S1.doc]

**EMECAR Study Group (in alphabetical order).**

Abasolo Alcazar L, Hospital Clínico Universitario San Carlos, Madrid

Alegre de Miguel C, Hospital de Malalties Reumatiques, Barcelona

Andreu Sánchez JL, Clínica Puerta de Hierro, Madrid

Aragón Díez A, Hospital Nuestra Señora del Prado, Talavera

Balsa Criado A, Hospital La Paz, Madrid

Batlle Gualda E, Hospital General Universitario de Alicante, Alicante

Belmonte Serrano MA, Hospital General de Castellón, Castellón

Beltrán Audera J, Hospital Clínico Universitario de Zaragoza, Zaragoza

Beltrán Fabregat J, Hospital General de Castellón, Castellón

Bonilla Hernan G, Hospital La Paz, Madrid

Carmona Ortells L, Fundación Española de Reumatología, Madrid

Caro Fernández N, Hospital Nuestra Señora del Prado, Talavera

Casado E, Hospital Universitario Germans Trias i Pujol, Badalona

Cebrian Mendez L, Hospital Gregorio Marañón, Madrid

Corteguera Coro M, Hospital Nuestra Señora de Sonsoles, Ávila

Cuadra Díaz JL, Hospital Nuestra Señora del Carmen, Ciudad Real

Cuesta E, Hospital Virgen de La Luz, Cuenca

Fiter Aresté J, Hospital Son Dureta, Mallorca

Freire Gonzalez M, Hospital Gregorio Marañón, Madrid

Galindo Izquierdo M, Hospital 12 de Octubre, Madrid

García Meijide JA, Hospital Clínico Universitario de Santiago, Santiago de Compostela

García Gómez MC, Hospital de Bellvitge Princeps D'Espanya, Barcelona

Giménez Ubeda E, Hospital Clínico Universitario de Zaragoza, Zaragoza

Gómez Centeno E, Hospital Clinic i Provincial, Barcelona

Gómez Vaquero C, Hospital de Bellvitge Princeps D'Espanya, Barcelona

González Fernández MJ, Hospital de Malalties Reumatiques, Barcelona

González Gómez ML, Hospital Gregorio Marañón, Madrid

González Hernández T, Instituto Provincial de Rehabilitación, Madrid

González-Alvaro I, Hospital de la Princesa, Madrid

González-Montagut Gómez C, Hospital Virgen de La Luz, Cuenca

Grandal Delgado Y, Hospital General de Jerez de La Frontera, Jerez

Gratacos Masmitja J, Complejo Hospitalario del Parc Tauli, Sabadell

Hernández del Río A, Hospital Juan Canalejo, La Coruña

Instxaurbe AR, Hospital de Basurto, Bilbao

Irigoyen Oyarzabal MV, Hospital General Carlos Haya, Málaga

Jiménez Palop M, Hospital Nuestra Señora de Sonsoles, Ávila

Juan Mas A, Hospital Son Llatzer, Mallorca

Júdez Navarro E, Hospital Clínico Universitario San Carlos, Madrid

Larrosa Padro M, Complejo Hospitalario del Parc Tauli, Sabadell

López Longo FJ, Hospital Gregorio Marañón, Madrid

Loza Santamaria E, Hospital Clínico Universitario San Carlos, Madrid

Maese Manzano J, Fundación Española de Reumatología, Madrid

Manero Ruiz FJ , Hospital Clínico Universitario de Zaragoza, Zaragoza

Mateo Bernardo I, Hospital 12 de Octubre, Madrid

Mayordomo González L, Hospital Universitario de Valme, Sevilla

Mazzucheli R, Hospital Fundación Alcorcón, Alcorcón

Medrano San Idelfonso M, Hospital Clínico Universitario de Zaragoza, Zaragoza

Naranjo Hernández A, Hospital de Gran Canaria Dr. Negrín, Gran Canaria

Pecondón Español A , Hospital Clínico Universitario de Zaragoza, Zaragoza

Peiró Callizo E, Hospital Virgen de La Luz, Cuenca

Quirós Donate J, Hospital Fundación Alcorcón, Alcorcón

Ramos López P, Hospital Príncipe de Asturias, Alcalá de Henares

Rivera Redondo J, Instituto Provincial de Rehabilitación, Madrid

Rodríguez Gómez M, Complejo Hospitalario Cristal-Piñor, Pontevedra

Rodríguez López M, Hospital Arquitecto Marcide, Pontevedra

Roselló Pardo R, Hospital General San Jorge, Huesca

Sampedro Alvarez J, Hospital Virgen de La Salud, Toledo

Sanmartí Sala R, Hospital Clinic i Provincial, Barcelona

Santos Rey Rey J, Hospital Virgen de La Salud, Toledo

Tena Marsá X, Hospital Universitario Germans Trias i Pujol, Badalona

Tenorio Martín M, Hospital del Insalud-Ceuta, Ceuta

Torres Martín MC, Hospital Nuestra Señora de Sonsoles, Ávila

Ureña Garnica I, Hospital General Carlos Haya, Málaga

Valdazo de Diego JP, Hospital General Virgen de La Concha, Zamora

Valls M, Hospital Universitario Germans Trias i Pujol, Badalona

Villaverde García V, Hospital La Paz, Madrid

Zarco Montejo P, Hospital Fundación Alcorcón, Alcorcón

Zubieta Tabernero J, Hospital Virgen de La Salud , Toledo
