# Supplementary material for: Trends towards an improved disease state in rheumatoid arthritis over time: influence of new therapies and changes in management approach: analysis of the EMECAR cohort
Source: Arthritis Res Ther. 2008 Nov 26;10(6):R138. doi: 10.1186/ar2561 (PMC2656242; doi:10.1186/ar2561)
Supplement: Additional file 2 — An Adobe file containing a figure that shows the flowchart of the EMECAR study, providing relevant information about the dropouts along the follow-up. [file ar2561-S2.pdf]

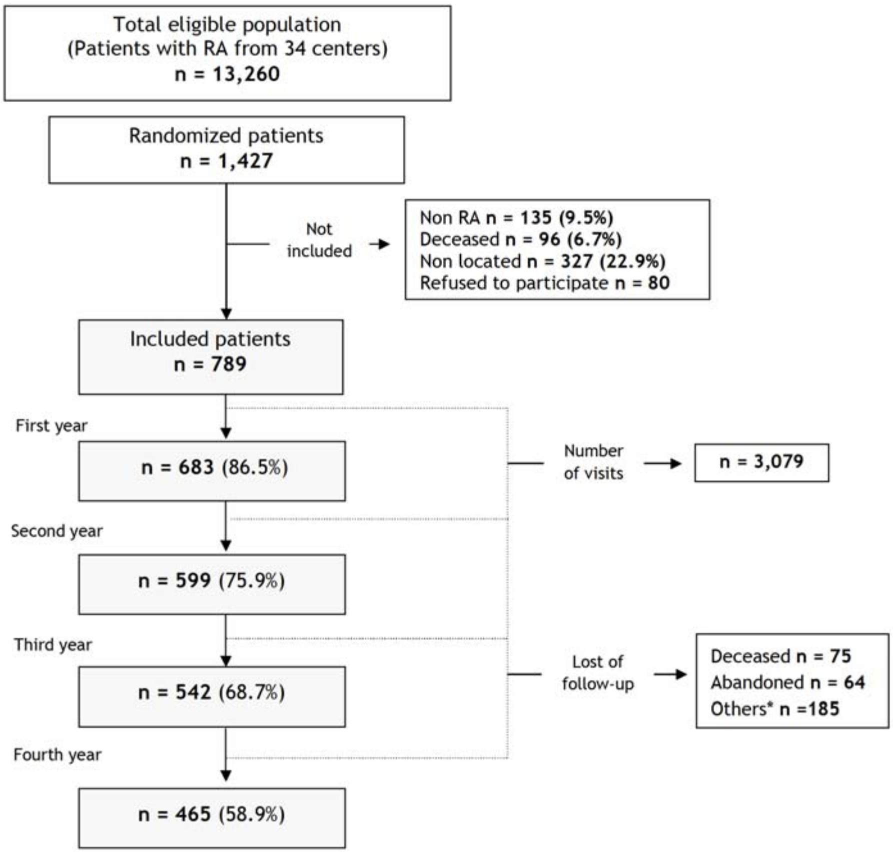

* Other reasons include: One center was closed during the study (n=10); 48 patients changed address or could not be located by their physicians despite repeated calls; due to a national rearrangement of specialists, some centers refused to continue in the study because the original investigator had left the center (n = 127).
