# Supplementary material for: Trends towards an improved disease state in rheumatoid arthritis over time: influence of new therapies and changes in management approach: analysis of the EMECAR cohort
Source: Arthritis Res Ther. 2008 Nov 26;10(6):R138. doi: 10.1186/ar2561 (PMC2656242; doi:10.1186/ar2561)
Supplement: Additional file 3 — A Word file containing a table that presents the characteristics of the studied patients and the nonstudied patients in the multivariable analysis of each variable: disease activity, functional disability and radiological damage. [file ar2561-S3.rtf]

Table 1. Characteristic of the patients studied and non-studied in the multivariable analysis of each variable: disease activity, functional disability and radiological damage.
 	DAS28	HAQ	Larsen	
 	Non-studied	Studied	Non-studied	Studied	Non-studied	Studied	
N	54	735	12	777	111	678	
Women, n (%)	40 (74)	528 (72)	9 (75)	559 (72)	84 (76)	484 (71)	
Age, mean ± SD	63 ± 11	61 ± 13	69 ± 10*	61 ± 13	62 ± 13	61 ± 13	
Age at RA onset, mean ± SD	48 ± 13	48 ± 15	56 ± 16	48 ± 15	49 ± 15	48 ± 14	
Rheumatoid factor positive, n (%)	34 (77)	544 (74)	3 (75)	575 (75)	71(70)	507 (75)	
Any comorbidity, n (%)	35 (65)*	571 (78)	2 (17)***	604 (78)	88 (79)	518 (76)	
Any extra-articular RA, n (%)	29 (54)	347 (47)	6 (50)	370 (48)	50 (45)	326 (48)	
BMI	27 ± 4.4	27 ± 4.3	28 ± 4.1	27 ± 4.3	27 ± 3.9	27 ± 4.4	
HAQ, mean ± SD	1.2 ± 0.9	1.2 ± 0.9	NA	1.2 ± 0.9	1.3 ± 0.9	1.2 ± 0.8	
DAS28, mean ± SD	NA	4.1 ± 1.4	4.7 ± 0.9	4.1 ± 1.4	4.1 ± 1.4	4.1 ± 1.4	
Larsen, mean ± SD	54 ± 20	55 ± 27	54 ± 11	54 ± 27	NA	54 ± 27	
		 		 		 	
***p<0.001;**p<0.01;*p<0.05;  	
Abbreviations: NA, not applicable.	
