# Supplementary figures and images for: Trends towards an improved disease state in rheumatoid arthritis over time: influence of new therapies and changes in management approach: analysis of the EMECAR cohort
Source: Arthritis Res Ther. 2008 Nov 26;10(6):R138. doi: 10.1186/ar2561 (PMC2656242; doi:10.1186/ar2561)

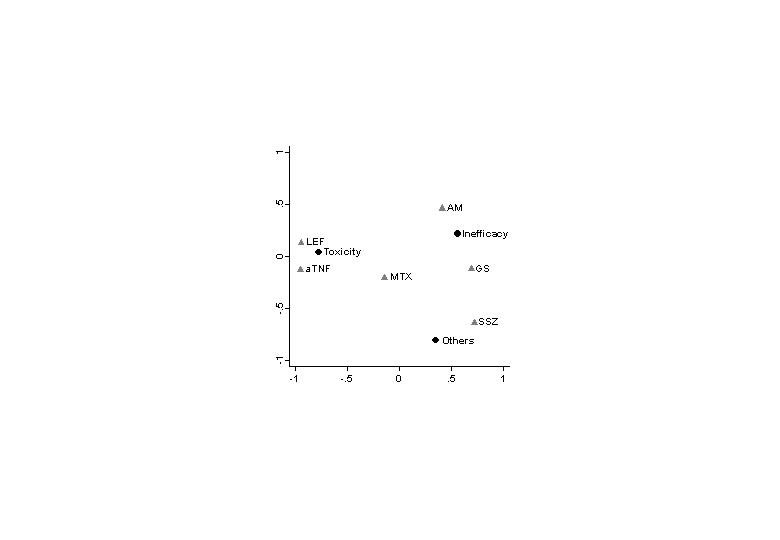

Supplement: Additional file 4 — An image file containing a graph of reasons for discontinuation during follow-up among therapies. The smaller the space between levels of different variables, the greater the association between them. AM, antimalarials; aTNF, TNF antagonists; GS, parenteral gold salts; LEF, leflunomide; MTX, methotrexate; SSZ, sulfasalazine. [file ar2561-S4.jpeg]
